# The Effect of Background Music in Shark Documentaries on Viewers' Perceptions of Sharks

**DOI:** 10.1371/journal.pone.0159279

**Published:** 2016-08-03

**Authors:** Andrew P. Nosal, Elizabeth A. Keenan, Philip A. Hastings, Ayelet Gneezy

**Affiliations:** 1 Center for Marine Biodiversity and Conservation, Scripps Institution of Oceanography, University of California San Diego, La Jolla, CA, United States of America; 2 Harvard Business School, Harvard University, Boston, MA, United States of America; 3 Marine Biology Research Division, Scripps Institution of Oceanography, University of California San Diego, La Jolla, CA, United States of America; 4 Rady School of Management, University of California San Diego, La Jolla, CA, United States of America; University of California Davis, UNITED STATES

## Abstract

Despite the ongoing need for shark conservation and management, prevailing negative sentiments marginalize these animals and legitimize permissive exploitation. These negative attitudes arise from an instinctive, yet exaggerated fear, which is validated and reinforced by disproportionate and sensationalistic news coverage of shark ‘attacks’ and by highlighting shark-on-human violence in popular movies and documentaries. In this study, we investigate another subtler, yet powerful factor that contributes to this fear: the ominous background music that often accompanies shark footage in documentaries. Using three experiments, we show that participants rated sharks more negatively and less positively after viewing a 60-second video clip of swimming sharks set to ominous background music, compared to participants who watched the same video clip set to uplifting background music, or silence. This finding was not an artifact of soundtrack alone because attitudes toward sharks did not differ among participants assigned to audio-only control treatments. This is the first study to demonstrate empirically that the connotative attributes of background music accompanying shark footage affect viewers’ attitudes toward sharks. Given that nature documentaries are often regarded as objective and authoritative sources of information, it is critical that documentary filmmakers and viewers are aware of how the soundtrack can affect the interpretation of the educational content.

## Introduction

Shark populations have declined worldwide due to overfishing, finning, and habitat degradation, with a quarter of these and related species now considered to be Threatened with extinction under IUCN criteria [1]. The urgent need for conservation and management notwithstanding, progress for sharks has been sluggish compared to, for example, marine mammals and sea turtles [2–4], which may be partially attributed to social marginalization of sharks that further legitimizes permissive exploitation of these animals [3]. Indeed, effective implementation and enforcement of conservation and management measures tightly hinges on public support. Despite evidence suggesting that changing perceptions of sharks has led to positive conservation impacts [5], gaining public support for shark conservation remains a challenge [6–7]. Thus, understanding the factors contributing to these negative attitudes is a vital part of the effort of conserving these animals.

Sharks have been vilified in human culture for centuries, and negative attitudes toward sharks continue to pervade mass media, perpetuating stereotypes, often conveying inaccurate information [7–11]. One way the public’s fear of sharks, which resonates deeply and viscerally, manifests itself is a pervasive overestimation of the likelihood of being ‘attacked’ [12]. For example, a sample of 766 Australians estimated that 7 to 9 fatal and 20 to 30 non-fatal shark bites occur every year in Australia, compared to the average of 1.1 fatal and 9.3 non-fatal bites per year that actually occurred between 1990 and 2010 [12].

The observed inflated fear of shark ‘attacks’ is driven, in part, by humans’ tendency to overweight the probability of rare events (e.g., shark bites, plane crashes, etc.). This tendency has been attributed to the availability heuristic by which risk judgments are based on the ease of recalling instances of such events [13–14], as well as on events’ memorability or imaginability, which are disproportionally salient in the context of extreme events [15]. Overestimation of risk can similarly be rooted in the dual-process nature of human reasoning and decision-making. In particular, because humans have limited resources for processing information, the tendency is to invoke our fast, instinctive, and emotional mental system first (i.e., System 1), and only activate our slower, more deliberative mental system (i.e., System 2) as needed [13, 16]. In the case of sharks, the associated instinctive, yet exaggerated fear is validated and reinforced by sensationalistic news coverage of shark ‘attacks’ [8–9] and by an emphasis on shark-on-human violence in shark documentaries [10, 17–18].

In this paper, we consider a subtler, yet powerful source of fear that has heretofore been overlooked: the ominous background music (*a la Jaws*) that often accompanies shark footage in documentaries. Music is ubiquitous and integral in film; it induces mood, communicates meaning, heightens the sense of reality, and enables symbolization [19–21]. Thus, the music accompanying shark footage is nontrivial. In fact, many people trace their fear of sharks to the 1975 blockbuster *Jaws*, whose redolent soundtrack has become deeply rooted in popular culture [17, 22]. *Jaws* epitomized the use of *leitmotif*, a short, recurring musical phrase that is continuously paired with a character such that eventually, the theme alone conjures up that character [23–24]. Just as the *leitmotif* of the Wicked Witch of the West from *The Wizard of Oz* might evoke images of its cackling, green-skinned character, the ominously quickening motif that typifies the *Jaws* soundtrack [24] may similarly evoke haunting images of surfacing dorsal fins, swimmers’ legs underwater, and the histrionic combination of blood and bubbles. Consequently, we propose that the background music in shark documentaries can negatively influence viewers’ perceptions of sharks, attitudes towards them, and likelihood of supporting related conservation efforts.

The emotional connotations of music are closely aligned with its structural attributes (e.g., key, interval, tempo, rhythm), and have been shown to influence individuals in a variety of ways [19–20, 25–26]. For example, in a study by Marshall and Cohen [27], subjects watched a short animated film of simple geometric shapes (a large triangle, a small triangle, and a small circle) moving within a rectangular enclosure, set to either fast-tempo or slow-tempo music. The soundtrack affected perceptions of the ‘characters’ in the film; for example, the large triangle was perceived as being more agitated and aggressive when the fast-tempo soundtrack played. Similarly, North [28] demonstrated that wine can taste like the music playing in the background. When a ‘zingy and fresh’ soundtrack played, tasters rated wine as significantly more ‘zingy and fresh’ than, say, ‘mellow and soft.’ Conversely, when a ‘mellow and soft’ soundtrack played in the background, tasters rated the same wine as significantly more ‘mellow and soft’ (see also Crisinel et al. [29]).

In this paper we hypothesized that documentary viewers’ perceptions of sharks would be greatly affected by the background music. In particular, we predicted that when footage of swimming sharks is set to ominous music, viewers would perceive sharks as scarier, more dangerous, and more vicious than when the same footage is set to uplifting music. Results from three experiments support our predictions: viewers perceive sharks more negatively (and less positively) after watching shark footage accompanied by ominous background music, versus uplifting background music or silence. This study is the first to investigate the impact of background music in shark documentary footage on viewers’ perceptions of sharks and, by extension, on their willingness to support related conservation efforts.

## Methods and Results

### Sample

The experiments were conducted online using Qualtrics. Respondents (*N* = 2181) were recruited using Amazon Mechanical Turk [30] and were paid US$0.25 or $0.50 for participating. All participants were from the United States and ranged in age from 18 to 73 years (*M*_age_ = 31.2, SD = 10.9 years); 953 (43.7%) were female (Goodness-of-Fit *χ*² = 34.42, df = 1, *p* < 0.001).

### Method and Design Overview

All experimental procedures described below were approved by the Institutional Review Board of the University of California–San Diego (Protocols #111362 and #130538). In all three experiments, participants were provided online informed consent. They were instructed to indicate their agreement to continue with the study by clicking forward. Those who agreed and subsequently passed an audio-visual equipment check were randomly assigned to one of six experimental treatments. Participants in the video treatments saw a 60-second video clip of sharks swimming, set either to uplifting music (V-uplifting), ominous music (V-ominous), or silence (V-silence). Participants in the audio-only treatments listened to the 60-second ominous (A-ominous) or uplifting audio clip alone (A-uplifting), or waited in silence for 60 seconds (A-silence). Participants in the video treatments were instructed to ‘watch the following documentary excerpt,’ while those in the A-uplifting and A-ominous treatments were instructed to ‘listen to the following musical excerpt.’ We informed participants in the A-silence treatment that ‘the next page takes approximately one minute to load’ and asked them to ‘wait patiently’ (see S1 Appendix for complete description of treatments). After completing this part of the experiment, participants answered a series of questions that measured their perceptions of sharks and willingness to conserve sharks (the questions varied slightly across experiments, and are described for each). Finally, participants indicated their gender, age, race/ethnicity, income, and political views. The complete dataset is provided in S1 Dataset.

### Stimulus Materials

The video clip used in all video treatments was an excerpt from the “Ocean World” episode of the *Blue Planet Seas of Life* series, which featured schooling requiem (Family Carcharhinidae) and hammerhead (Family Sphyrnidae) sharks swimming innocuously. This video clip was set to either uplifting background music, ominous background music, or silence. The ominous background music was an excerpt from Track 8 (“Sharks”) of the *Blue Planet*: *Music from the BBC TV Series* soundtrack. This clip was assessed by an independent music expert blind to the objectives and nature of the study, who described it as ‘modal with only fragments of melody’ accompanied by ‘sporadic and sparse atmospheric percussion’ and ‘a repetitive flute motif that creates an unsettling sound,’ thus confirming the ominous nature of the music. The uplifting background music was an excerpt from Track 1 (“The Blue Planet”) of the same soundtrack and was evaluated by the same music expert who confirmed its uplifting nature (see S1 Appendix for complete description of stimulus materials).

### Experiment 1

Data for Experiment 1 were collected from 14 to 21 August 2013. A total of 616 individuals (*M*_age_ = 30.2, SD = 10.3 years; 39.6% females; Goodness-of-Fit *χ*² = 26.18, df = 1, *p* < 0.001) participated and were paid US$0.25. The raw sample included 636 participants of which 20 were dropped because they failed the audio-visual equipment check (*N* = 9) or did not complete the survey (*N* = 11) for undetermined reasons (see S1 Appendix for complete sample demographics).

#### Perception Measure

To measure their attitudes toward sharks, participants were asked to indicate to what extent they thought each of six words capturing negative (scary, dangerous, vicious) and positive (peaceful, beautiful, graceful) associations describe sharks. Participants rated each adjective on a 7-point scale ranging from 1 (*not at all*) to 7 (*very much*). Finally, we asked participants to ‘write one additional word in the space below, other than those listed above, that you would use to describe sharks.’

#### Willingness-to-Conserve Measure

Next, to measure their willingness to support efforts to conserve sharks, we asked participants to indicate ‘to what extent do you support measures to restore depleted shark populations (such as banning or regulating shark fishing, establishing no-fishing reserves, etc.), effectively increasing the number of sharks in the ocean?’ on a 7-point scale ranging from 1 (*not at all*) to 7 (*very much*).

#### Results

A factor analysis with varimax (orthogonal) rotation was conducted on participants’ ratings of the six adjectives. The sample was determined to be factorable by its Kaiser-Meyer-Olkin (KMO) value of 0.823 [31–32]. The analysis yielded a two-factor solution with simple structure; scary, dangerous, and vicious loaded onto Factor 1 (labeled *negative*) with loadings of 0.858, 0.894, and 0.851, respectively; peaceful, beautiful, and graceful loaded onto Factor 2 (labeled *positive*) with loadings of 0.642, 0.891, and 0.891, respectively.

**Fig 1:**
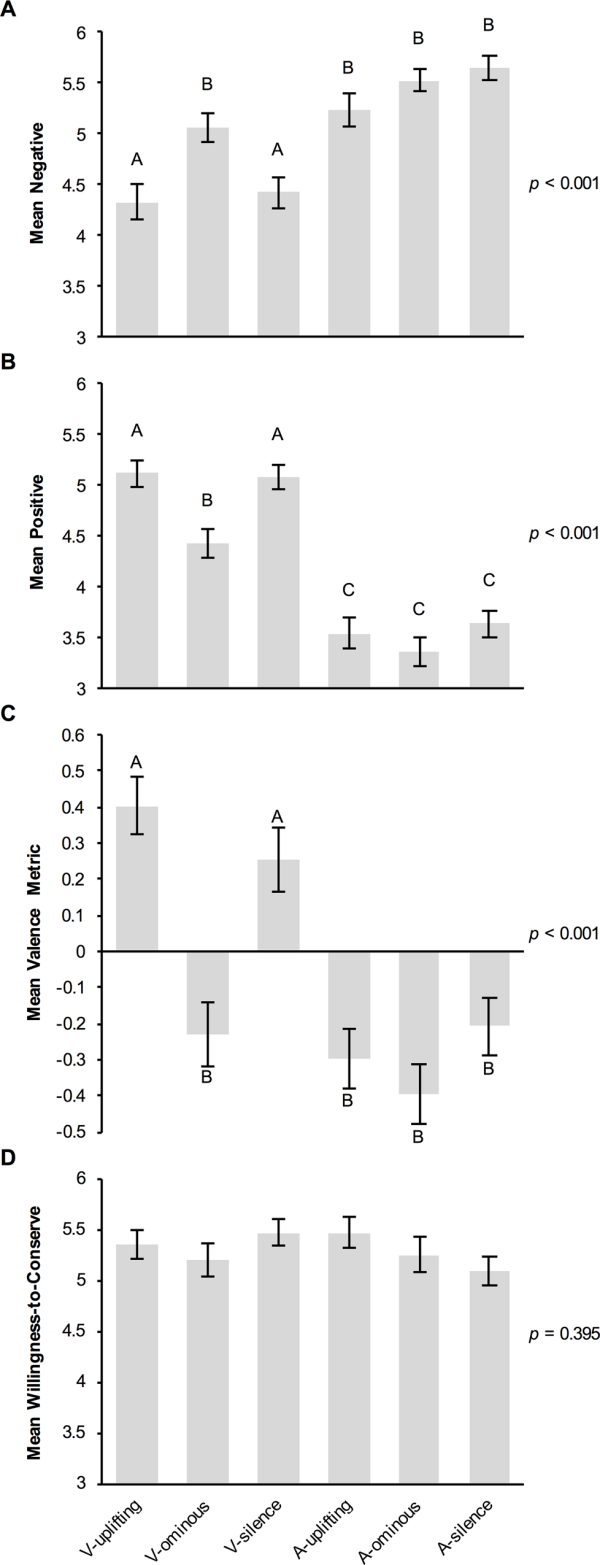
Effects of experimental treatment on measures of perception of and willingness to conserve sharks (Experiment 1). (A) Mean *negative* and (B) *positive* ratings of sharks, derived from participants’ raw ratings of how well each of three negative adjectives (scary, dangerous, vicious) and three positive adjectives (peaceful, graceful, beautiful) describe sharks (1 = *not at all*, 7 = *very much*). (C) Mean valence of one additional adjective provided by participants to describe sharks (-1 = *negative*, 1 = *positive*). (D) Mean willingness to support ‘measures to restore depleted shark populations’ (1 = *not at all*, 7 = *very much*). All means are given with ± SEM. Letters indicate the results of post hoc Dunn’s *Z* Tests performed for all 15 pairwise comparisons if the overall Kruskal-Wallis Test was significant (A, B, C); means with the same letter are not significantly different from each other (i.e., Bonferroni-adjusted *p* > 0.05).

There was a significant effect of experimental treatment on both negative (Kruskal-Wallis *H* = 62.423, df = 5, *p* < 0.001) and positive (*H* = 140.523, df = 5, *p* < 0.001; Fig 1) ratings of sharks. Participants in the V-ominous treatment rated sharks significantly more negatively (*M* = 5.07) than those in the V-uplifting (*M* = 4.33; Dunn’s *Z* = 2.993, Bonferroni-adjusted *p* = 0.042) and V-silence treatments (*M* = 4.43; *Z* = 3.023, *p* = 0.038). Similarly, participants in the V-ominous treatment rated sharks significantly less positively (*M* = 4.43) than those in the V-uplifting (*M* = 5.11; *Z* = 3.534, *p* = 0.006) and V-silence (*M* = 5.08; *Z* = 3.240, *p* = 0.018) treatments. There was no significant difference in either positive or negative ratings of sharks between the V-uplifting and V-silence treatments (*Z* = 0.147–0.286, *p* = 1). Conversely, there was no significant difference in participants’ negative (*M* = 5.23–5.65; *Z* = 0.266–1.244, *p* = 1) or positive ratings (*M* = 3.36–3.64; *Z* = 0.156–0.497, *p* = 1) among the audio-only treatments, ruling out the possibility that the effect is merely driven by the music itself. Finally, there was no significant effect of treatment on participants’ willingness to support shark conservation (*H* = 5.175, df = 5, *p* = 0.395; Fig 1).

Two research assistants, blind to the experimental objectives and design, independently coded participants’ free-response adjectives as either *positive*, *negative*, *neutral*, or *unknown*. Fourteen responses were omitted from the analysis due to participants not following instructions (e.g., response was unintelligible or consisted of several words instead of one as instructed). Intercoder agreement was 86.4% (Cohen’s κ = 0.797, 95% CI: 0.758–0.836, *p* < 0.001), however, after discussion intercoder agreement increased to 99.7%. Two additional responses were omitted due to intercoder deadlock. A simple valence metric was calculated by coding *positive* adjectives as 1, *negative* adjectives as -1, and *neutral* adjectives as 0 (*unknown* adjectives were omitted; *N* = 22). A Kruskal-Wallis test revealed a significant effect of experimental treatment on valence (*H* = 66.367, df = 5, *p* < 0.001; Fig 1), with participants in the V-ominous treatment providing adjectives with more negative valence (*M* = -0.229) than those in the V-uplifting (*M* = 0.404; Dunn’s *Z* = 5.079, Bonferroni-adjusted *p* < 0.001) and V-silence (*M* = 0.253; *Z* = 3.838, *p* = 0.002; Fig 1) treatments. There was no significant difference in valence between the V-uplifting and V-silence treatments (*Z* = 1.250; *p* = 1). There was also no significant difference in valence among the audio-only treatments (*M* = -0.396–-0.206; *Z* = 0.726–1.542, *p* = 1).

#### Discussion

The findings of Experiment 1 show that viewer perceptions are greatly influenced by the background music accompanying shark footage. Participants who viewed a video clip set to ominous music rated sharks more negatively and less positively than those who watched the same clip set to uplifting music or to silence. This result is not an artifact of the soundtrack alone because there were no differences in the ratings of participants among the three audio-only treatments. Finally, despite the effect of background music on perception of sharks, participants’ willingness to support conservation efforts was not affected. Although speculative, it is possible that the effect of the treatment stimulus dissipated by the time participants completed this measure. To address this potential concern, participants in Experiment 2 completed the willingness-to-conserve measure immediately after being exposed to the experimental treatment, followed by the perception measure. Additionally, to test whether the lack of effect was due to the particular willingness-to-conserve measure used, we presented some participants in Experiment 2 with a different willingness-to-conserve measure.

### Experiment 2

Data for Experiment 2 were collected from 27 to 30 August 2013. A total of 806 individuals (*M*_age_ = 32.0, SD = 11.0 years; 40.4% females; Goodness-of-Fit *χ*² = 29.04, df = 1, *p* < 0.001) participated and were paid US$0.50. The raw sample included 831 participants of which 25 were dropped because they failed the audio-visual equipment check (*N* = 18) or did not complete the survey (*N* = 7) for undetermined reasons (see S1 Appendix for complete sample demographics). Participants in Experiment 2 were randomly assigned to one of two possible willingness-to-conserve measures (designated 2a and 2b), which were presented immediately after the experimental treatment, followed by the same perception measure used in Experiment 1.

About half of participants (*N* = 404) were randomly assigned to the same willingness-to- conserve measure used in Experiment 1 (2a), whereas the rest (*N* = 402) were asked ‘how much would you be willing to donate to ‘a non-profit organization whose mission includes protecting sharks and increasing shark populations around the world?’ Participants indicated their hypothetical donation using a slider scale ranging from US$0 to US$100 in increments of US$1 (2b). The results are herein reported in two parts (2a and 2b), broken out based on the two willingness-to-conserve measures.

#### Results

**Fig 2:**
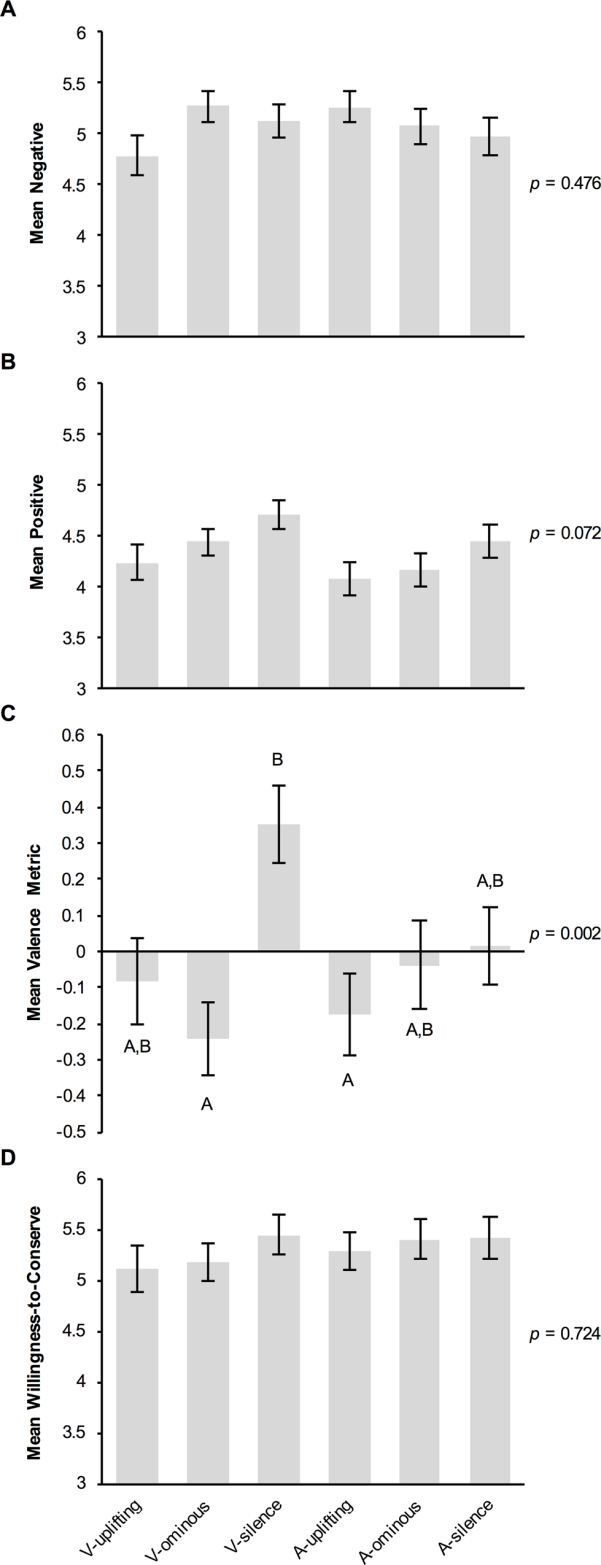
Effects of experimental treatment on measures of perception of and willingness to conserve sharks (2a). (A) Mean *negative* and (B) *positive* ratings of sharks, derived from participants’ raw ratings of how well each of three negative adjectives (scary, dangerous, vicious) and three positive adjectives (peaceful, graceful, beautiful) describe sharks (1 = *not at all*, 7 = *very much*). (C) Mean valence of the adjective provided by participants to describe sharks (-1 = *negative*, 1 = *positive*). (D) Mean willingness to support ‘measures to restore depleted shark populations’ (1 = *not at all*, 7 = *very much*). All means are given with ± SEM. Letters indicate the results of post hoc Dunn’s *Z* Tests performed for all 15 pairwise comparisons if the overall Kruskal-Wallis Test was significant (C); means with the same letter are not significantly different from each other (i.e., Bonferroni-adjusted *p* > 0.05).

*Willingness to Support Conservation Efforts (2a)*: Again, there was no significant effect of treatment on willingness to support ‘measures to restore depleted shark populations’ (Kruskal-Wallis *H* = 2.845, df = 5, *p* = 0.724; Fig 2). The sample of adjective ratings was determined to be factorable by its KMO value of 0.746. As in Experiment 1, the analysis yielded a two-factor solution with simple structure; scary, dangerous, and vicious loaded onto Factor 1 (labeled *negative*) with loadings of 0.862, 0.878, and 0.858, respectively; peaceful, beautiful, and graceful loaded onto Factor 2 (labeled *positive*) with loadings of 0.652, 0.871, and 0.853, respectively. However, there was no significant effect of treatment on negative (Kruskal-Wallis *H* = 4.527, df = 5, *p* = 0.476; Fig 2) or positive perceptions of sharks (*H* = 10.134, df = 5, *p* = 0.072; Fig 2).

In the process of coding the free-response adjectives to describe sharks, 8 of the 404 responses were omitted due to participants not following instructions. Intercoder agreement was 91.1% (Cohen’s κ = 0.851, 95% CI: 0.808–0.894, *p* < 0.001), however, after discussion intercoder agreement increased to 99.3%. Three additional responses were omitted due to intercoder deadlock. There was a significant effect of experimental treatment on the valence metric derived from these coding results (*H* = 17.975, df = 5, *p* = 0.003; 18 unknown adjectives were omitted; Fig 2), with participants in the V-ominous treatment providing adjectives that had significantly more negative valence (*M* = -0.243) than those in the V-silence treatment (*M* = 0.354; Dunn’s *Z* = 3.896, Bonferroni-adjusted *p* = 0.001). There was no significant difference in valence between the V-uplifting (*M* = -0.083) and the V-ominous or V-silence treatments (Z = 1.019–2.710, *p* = 0.101–1). There was also no significant difference in valence among the audio-only treatments (*M* = -0.177–0.017; *Z* = 0.225–0.735, *p* = 1).

**Fig 3:**
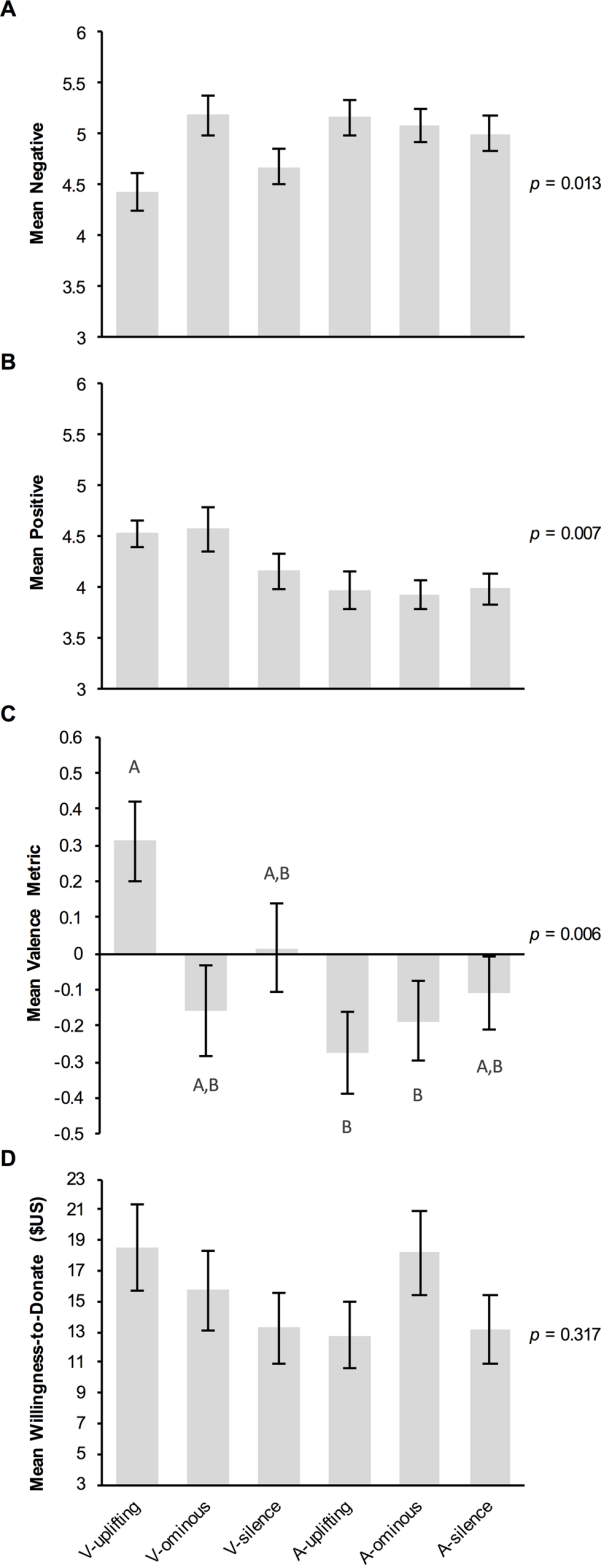
Effects of experimental treatment on measures of perception of and willingness to donate (2b). (A) Mean *negative* and (B) *positive* ratings of sharks, derived from participants’ raw ratings of how well each of three negative adjectives (scary, dangerous, vicious) and three positive adjectives (peaceful, graceful, beautiful) describe sharks (1 = *not at all*, 7 = *very much*). (C) Mean valence of one additional adjective provided by participants to describe sharks (-1 = *negative*, 1 = *positive*). (D) Mean willingness to donate to ‘a non-profit organization whose mission includes protecting sharks and increasing shark populations around the world’ (US$1 increments from US$0 to US$100). All means are given with ± SEM. Letters indicate the results of post hoc Dunn’s *Z* Tests performed for all 15 pairwise comparisons if the overall Kruskal-Wallis Test was significant (C); means with the same letter are not significantly different from each other (i.e., Bonferroni-adjusted *p* > 0.05).

*Willingness to Donate (2b)*: There was no effect of treatment on participants’ willingness to donate (*H* = 5.891, df = 5, *p* = 0.317; Fig 3). The sample of adjective ratings was determined to be factorable by its KMO value of 0.786. The analysis yielded a two-factor solution with simple structure; scary, dangerous, and vicious loaded onto Factor 1 (labeled *negative*) with loadings of 0.828, 0.885, and 0.865, respectively; peaceful, beautiful, and graceful loaded onto Factor 2 (labeled *positive*) with loadings of 0.678, 0.889, and 0.861, respectively. Although there was a significant overall effect of treatment on negative (Kruskal-Wallis *H* = 14.400, df = 5, *p* = 0.013) and positive ratings of sharks (*H* = 15.927, df = 5, *p* = 0.007), none of the post hoc pairwise comparisons were significant after adjusting for multiple comparisons (Fig 3).

In the process of coding the free-response adjectives to describe sharks, 11 of the 402 responses were omitted due to participants not following instructions. Intercoder agreement was 95.3% (Cohen’s κ = 0.935, 95% CI: 0.906–0.964, *p* < 0.001), however after discussion intercoder agreement increased to 98.8%. Five additional responses were omitted due to intercoder deadlock. Although there was an overall effect of experimental treatment on the valence metric derived from these coding results (*H* = 16.184, df = 5, *p* = 0.006; 26 unknown adjectives were omitted; Fig 3), this was driven by participants in the V-uplifting treatment providing adjectives that had significantly more positive valence (*M* = 0.311) than those in the A-uplifting (*M* = -0.276; Dunn’s *Z* = 3.567, Bonferroni-adjusted *p* = 0.005) and A-ominous treatments (*M* = -0.188; *Z* = 3.100, *p* = 0.029). There was no significant difference in valence among the three video treatments (*M* = -0.160–0.311; *Z* = 0.318–2.743, *p* = 0.091–1) or among the three audio-only treatments (*M* = -0.276–-0.108; *Z* = 0.275–0.578, *p* = 1).

#### Discussion

The results of Experiment 2 suggest that a dissipation effect of the treatment stimulus does not likely explain the null effect observed on the willingness-to-conserve measure in Experiment 1. In contrast, it appears that the treatment effect on participants’ perceptions of sharks may actually dissipate over time, recalling that in this experiment, the perception measure followed the willingness-to-conserve measure. Although the general trends resemble the results observed in Experiment 1, the effects are weaker and ambiguous with respect to the underlying source (Figs 2 and 3). Lastly, neither willingness-to-conserve measure was affected by treatment in Experiment 2. Prior work suggests that willingness to donate toward the conservation of sharks is strongly influenced by a number of factors including levels of concern for sharks [33]. While it is plausible that individuals’ unwillingness to help protect sharks is rooted in concerns or beliefs that run too deep to be altered by the short, one-time stimuli used in our experiments, it is also possible that the hypothetical nature of our measures made it difficult to detect an effect. To address this potential concern, in Experiment 3 participants’ decisions involved actual donations. Moreover, we sought to replicate the effect of music on perceptions obtained in Experiment 1.

### Experiment 3

Data for Experiment 3 were collected from 16 December 2014 to 21 February 2015. A total of 759 individuals (*M*_age_ = 33.2, SD = 11.0 years; 50.5% females; Goodness-of-Fit *χ*² = 0.04, df = 1, *p* = 0.842) participated and were paid US$0.25. The raw sample included 796 participants of which 37 were dropped because they failed the audio-visual equipment check (*N* = 25) or did not complete the survey (*N* = 12) for undetermined reasons (see S1 Appendix for complete sample demographics). We captured participants’ perceptions of sharks with the same measure used in Experiment 1. Also similar to Experiment 1, the perception measure was taken immediately after the experimental treatment. Next, we informed participants that at the conclusion of the study, the researchers would make a US$100 donation to ‘a non-profit organization dedicated to protecting and restoring the world’s oceans,’ designated in its entirety to one of three possible funds: ‘protecting sharks,’ ‘protecting dolphins,’ or ‘discretionary fund.’ Participants were asked to vote for which fund should receive the donation (the authors indeed made a donation of US$100 to this organization, which they designated to the fund with the most votes).

#### Results

The sample of adjective ratings was determined to be factorable by its KMO value of 0.813. As in Experiments 1 and 2, the analysis yielded a two-factor solution with simple structure; scary, dangerous, and vicious loaded onto Factor 1 (labeled *negative*) with loadings of 0.886, 0.888, and 0.859, respectively; peaceful, beautiful, and graceful loaded onto Factor 2 (labeled *positive*) with loadings of 0.657, 0.895, and 0.895, respectively.

**Fig 4:**
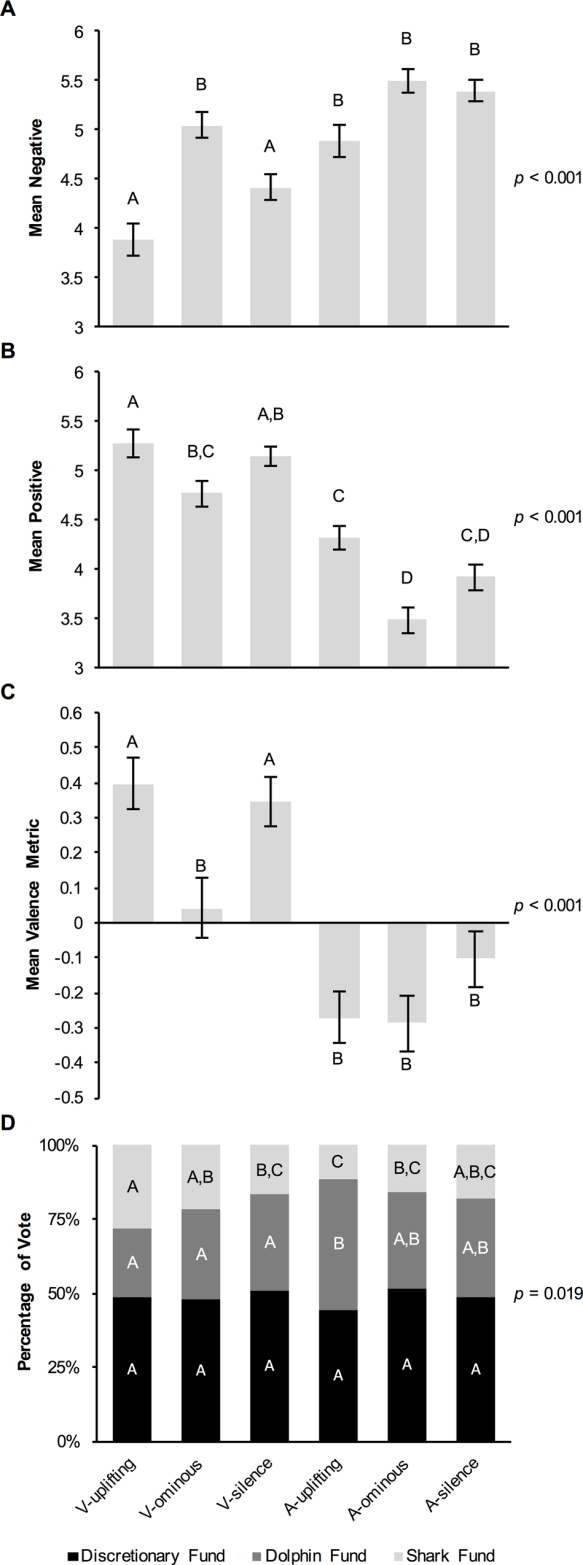
Effects of experimental treatment on measures of perception of and willingness to conserve sharks (Experiment 3). (A) Mean *negative* and (B) *positive* ratings of sharks, derived from participants’ raw ratings of how well each of three negative adjectives (scary, dangerous, vicious) and three positive adjectives (peaceful, graceful, beautiful) describe sharks (1 = *not at all*, 7 = *very much*). (C) Mean valence of one additional adjective provided by participants to describe sharks (-1 = *negative*, 1 = *positive*). (D) Percentage of participants choosing to allocate an existing donation of US$100 to each of three funds (Shark Fund, Dolphin Fund, Discretionary Fund) at ‘a non-profit organization dedicated to protecting and restoring the world’s oceans.’ All means are given with ± SEM. Letters indicate the results of post hoc Dunn’s *Z* Tests performed for all 15 pairwise comparisons if the overall Kruskal-Wallis Test was significant (A, B, C); means with the same letter are not significantly different from each other (i.e., Bonferroni-adjusted *p* > 0.05). Similarly, letters in D indicate the results of post hoc pairwise *Z* tests on the proportions of participants choosing each fund across the six treatments.

There was a significant effect of experimental treatment on both negative (Kruskal-Wallis *H* = 80.789, df = 5, *p* < 0.001; Fig 4) and positive (*H* = 131.803, df = 5, *p* < 0.001; Fig 4) perceptions of sharks. Participants in the V-ominous treatment rated sharks significantly more negatively (*M* = 5.04) than those in the V-uplifting (*M* = 3.88; Dunn’s *Z* = 4.855, Bonferroni-adjusted *p* < 0.001) and V-silence treatments (*M* = 4.41; *Z* = 3.206, *p* = 0.020). Similarly, participants in the V-ominous treatment rated sharks significantly less positively (*M* = 4.77) than those in the V-uplifting treatment (*M* = 5.27; *Z* = 2.945, *p* = 0.048). There was no difference in positive rating between the V-ominous and V-silence treatments (*M* = 5.15; *Z* = 1.875, *p* = 0.913). There was also no significant difference in either positive or negative ratings of sharks between the V-uplifting and V-silence treatments (*Z* = 0.952–1.649, *p* = 1). Finally, there was no significant difference in negative rating (*M* = 4.88–5.50; *Z* = 1.022–2.557, *p* = 0.159–1) among the audio-only treatments, however positive rating was lower in the A-ominous treatment (*M* = 3.49) compared to the A-uplifting treatment (*M* = 4.32; *Z* = 4.192, *p* < 0.001).

During the process of coding the free-response adjectives to describe sharks, 21 of the 759 responses were omitted due to participants not following instructions. Intercoder agreement was 99.9% (Cohen’s κ = 0.998, 95% CI: 0.994–1.000, *p* < 0.001), however, after discussion intercoder agreement reached 100%. There was a significant effect of experimental treatment on the valence metric derived from these coding results (*H* = 66.487, df = 5, *p* < 0.001; 26 unknown adjectives were omitted; Fig 4), with participants in the V-ominous treatment providing adjectives that had significantly more negative valence (*M* = 0.042) than those in the V-uplifting (*M* = 0.397; Dunn’s *Z* = 3.075, Bonferroni-adjusted *p* = 0.032) and V-silence (*M* = 0.347; *Z* = 2.955, *p* = 0.047) treatments. There was no significant difference in valence between the V-uplifting and V-silence treatments (*Z* = 0.442, *p* = 1) or among the three audio-only treatments (*M* = -0.286–-0.103; *Z* = 0.129–1.550, *p* = 1).

Lastly, there was a significant effect of treatment on participants’ choice of fund (‘protecting sharks,’ ‘protecting dolphins,’ or ‘discretionary fund’) to receive a US$100 donation (Pearson *χ*² = 21.286, df = 10, *p* = 0.019; Fig 4). A significantly higher proportion of participants chose to protect sharks in the V-uplifting treatment (0.280) than in the V-silence treatment (0.168; *Z* = 2.124, *p* = 0.034). However, there was no significant difference in the proportion of participants choosing to protect sharks between the V-uplifting and V-ominous (0.216) treatments (*Z* = 1.172, *p* = 0.242), or the V-ominous and V-silence treatments *(Z* = 0.963, *p* = 0.337). Consistent with the results obtained thus far, there was no significant difference in the proportion of participants choosing to protect sharks (0.112–0.181; *Z* = 0.563–1.587, *p* = 0.114–0.575) among the three audio-only treatments.

#### Discussion

The findings of Experiment 3 are consistent with those of Experiment 1, showing that participants regarded sharks more negatively and less positively after watching a shark video clip set to ominous, versus to uplifting music or to silence. Unlike Experiments 1 and 2, we found a significant effect of treatment on behavior: participants were more likely to allocate funds to protecting sharks after viewing the shark video set to uplifting music than when the video was set to silence. Even though the behavioral effect is limited to the comparison of the V-uplifting and V-silence treatments, it offers some support to the proposed role of background music on behavior, and suggests further research is warranted.

## Conclusions

The current study is the first to demonstrate empirically that the soundtrack accompanying shark documentary footage can affect viewers’ perceptions of sharks. Participants who viewed a 60-second video clip of swimming sharks set to ominous background music regarded sharks more negatively and less positively than those who watched the same video clip set to uplifting background music or to silence. Notably, participants who did not watch the video clip, but only listened to the 60-second uplifting or ominous audio clip (or waited in silence for 60 seconds), generally regarded sharks more negatively and less positively than those who watched the video clip. In the absence of any visual stimulus, these sentiments may reflect individuals’ baseline attitudes towards sharks. For example, participants in the audio-only treatments across all three experiments rated sharks significantly more negatively (*M* = 5.251) than those in the video treatments (*M* = 4.665; Mann-Whitney *U* = 725505, *p* < 0.001). Similarly, positive ratings were significantly lower in the audio-only treatments (*M* = 3.870) compared to the video treatments (*M* = 4.781; *U* = 383753, *p* < 0.001). Although the comparison between video and audio-only treatments was not the primary intent of this study, the results are nevertheless interesting and presented separately for each of the three experiments in S1 Appendix.

Whereas the background music accompanying shark footage affects viewers’ perceptions of sharks, it remains unclear whether this attitude shift would also influence behavior. Only in Experiment 3 did we observe a treatment effect on willingness to support shark conservation; although this result is compelling, it may not necessarily reflect the public’s willingness to engage in pro-conservation behavior per se, but rather may be an artifact of the specific measure used (i.e., designation of resources already allocated to conservation).

It has already been shown that engaging a supportive public in shark conservation is challenging due to generally negative attitudes toward sharks [6–7]. For many, documentaries are regarded as objective and authoritative sources of information [34], and for some, documentaries may in fact be the primary source of information on animals such as sharks. Thus, documentary filmmakers and viewers should be aware of the effects of the soundtrack on the interpretation of the educational content. Similar consideration should be given in the production of news packages and curation of shark exhibits. Filmmakers, journalists, and exhibit designers set the tone of their works, and, while an ominous soundtrack may enhance their entertainment aspect, it may also undermine their educational value by biasing viewers’ perceptions of sharks. This, in turn, may impede legitimate shark conservation efforts and fuel counterproductive management programs like culling and setting shark nets. Most importantly, this study specifically highlights the need to raise the public’s awareness of the effect of background music in shark documentaries in the hope that it would decrease the extent by which they are affected by it.

## Supporting Information

S1 Appendix Additional details on experimental treatments, stimulus materials, sample demographics, and analyses on perception measures between video and audio-only treatments. (DOCX)

S1 Dataset Spreadsheet containing the complete dataset for Experiments 1, 2a, 2b, and 3. (XLSX)
