# Supplementary material for: The Effect of Background Music in Shark Documentaries on Viewers' Perceptions of Sharks
Source: PLoS One. 2016 Aug 3;11(8):e0159279. doi: 10.1371/journal.pone.0159279 (PMC4972383; doi:10.1371/journal.pone.0159279)
Supplement: S1 Appendix — (DOCX) [file pone.0159279.s001.docx]

**Appendix**

**Experimental Treatments**

After consenting to participate and passing an audio-visual equipment check, participants in all three experiments were randomly assigned to one of six experimental treatments. These included viewing a 60-second video clip of sharks swimming, set either to uplifting music (V-uplifting), ominous music (V-ominous), or silence (V-silence). In the remaining three treatments, participants only listened to the 60-second ominous (A-ominous) or uplifting audio clip alone (A-uplifting), or waited in silence for 60 seconds (A-silence). For the three video treatments (V-ominous, V-uplifting, V-silence), participants were instructed to ‘watch the following documentary excerpt’ with the video clip embedded below the prompt. Participants in the audio conditions (A-ominous, A-uplifting), were instructed to ‘listen to the following musical excerpt’ with the audio clip embedded below the prompt. Lastly, we advised those in the third audio condition (A-silence) that ‘the next page takes approximately one minute to load’, and instructed them to ‘wait patiently’ with a 60-second countdown timer embedded below the prompt. The countdown timer was used to give participants in the A-silence condition a similar sense of elapsed time to participants in the other conditions, who could monitor the progress bar associated with their 60-second video or audio clips.

**Stimulus Materials**

**Video**

The video clip used was a 60-second excerpt from the “Ocean World” episode of the *Blue Planet Seas of Life* series (Original UK Version, 2001 British Broadcasting Corporation; 10:50 – 11:50 of video track), which featured schooling requiem (Family Carcharhinidae) and hammerhead (Family Sphyrnidae) sharks swimming innocuously. This video clip was set to either uplifting background music, ominous background music, or silence.

**Audio**

The ominous background music was a 60-second excerpt from Track 8 (“Sharks”) of the *Blue Planet: Music from the BBC TV Series* soundtrack (2002 George Fenton, Koch Records; 1:45 – 2:45 of audio track). We chose this track because it closely resembled the background music of the original video clip from the *Blue Planet Seas of Life* episode. This clip was assessed by an independent music expert blind to the objectives and nature of this study. The expert concluded the following:

“This clip initially features sustained low-range string textures in E Phrygian with half-step trills complementing the drone before it modulates down to E-flat. Once in E-flat, sporadic and sparse atmospheric percussion is combined with a repetitive flute motif that creates an unsettling sound over which the low strings outline a diminished chord, which is unstable to begin with. Overall, this clip is atmospheric and modal with only fragments of melody.”

The uplifting background music was a 60-second excerpt from Track 1 (“The Blue Planet”) of the the same soundtrack (1:41 – 2:41 of audio track). The same music expert mentioned above assessed this clip as well and concluded the following:

“This clip is largely diatonic and mostly in a major key, featuring a clear melody comprised of similar motifs that repeat over a strongly harmonic background. There is brief upward modulation that creates a sense of lifting. As with the previous clip, this one also features low strings, but uses them harmonically rather than modally. Finally, the timbre of brass and the human voice create a strong, bright sound.”

Because the stimulus materials (i.e., video and audio clips) contain copyrighted material, they could not be published with our manuscript under the Creative Commons Attribution License (CCAL), CC BY 4.0; however, they are available from the authors upon request.

**Experiment 1 Sample Demographics**

Data for Experiment 1 were collected from 14 to 21 August 2013. A total of 616 individuals (*M*_age_ = 30.2, SD = 10.3 years; 39.6% females; Goodness-of-Fit *χ*² = 26.18, df = 1, *p* < 0.001) participated and were paid US$0.25. The raw sample included 636 participants of which 20 were dropped because they failed the audio-visual equipment check (*N* = 9) or did not complete the survey for undetermined reasons (*N* = 11). There was no significant difference in participants’ gender (Pearson *χ*² = 1.14, df = 5, *p* = 0.950), age (Kruskal-Wallis *H* = 2.973, df = 5, *p* = 0.704), race/ethnicity (Fisher Exact *p* = 0.912), political views (Fisher Exact *p* = 0.104), or annual income (Pearson *χ*² = 13.697, df = 20, *p* = 0.846) across conditions.

**Experiment 2 Sample Demographics**

Data for Experiment 2 were collected from 27 to 30 August 2013. A total of 806 individuals (*M*_age_ = 32.0, SD = 11.0 years; 40.4% females; Goodness-of-Fit *χ*² = 29.04, df = 1, *p* < 0.001) participated and were paid US$0.50. The raw sample included 831 participants of which 25 were dropped because they failed the audio-visual equipment check (*N* = 18) or did not complete the survey for undetermined reasons (*N* = 7). Participants in Experiment 2 were randomly assigned to one of two possible *willingness-to-conserve measures*, which were presented immediately after the experimental manipulation treatment, followed by the same *perception measure* used in Experiment 1.

One of the *willingness-to-conserve measures* (2a) was identical to Experiment 1. A total of 404 individuals (*M*_age_ = 31.8, SD = 10.9 years; 40.8% females; Goodness-of-Fit *χ*² = 13.2, df = 1, *p* < 0.001) were randomly assigned to this measure, with no significant difference in age (Kruskal-Wallis *H* = 6.924, df = 5, *p* = 0.226), gender (Pearson *χ*² = 3.391, df = 5, *p* = 0.643), race/ethnicity (Fisher Exact *p* = 0.545), political views (Fisher Exact *p* = 0.751), or annual income (Fisher Exact *p* = 0.860) across conditions.

In the other *willingness-to-conserve measure* (2b), we asked participants ‘how much would you be willing to donate’ to ‘a non-profit organization whose mission includes protecting sharks and increasing shark populations around the world?’ Participants indicated their hypothetical donation using a slider scale ranging from US$0 to US$100 in increments of US$1. A total of 402 individuals (*M*_age_ = 32.2, SD = 11.1; 40.0% females; Goodness-of-Fit *χ*² = 15.52, df = 1, *p* < 0.001) were randomly assigned to this measure with no significant difference in race/ethnicity (Fisher Exact *p* = 0.468), political views (Fisher Exact *p* = 0.423), or annual income (Fisher Exact *p* = 0.344) across conditions. Although there were overall differences in age (Kruskal-Wallis *H* = 18.947, df = 5, *p* = 0.002) and gender (Pearson *χ*² = 19.510, df = 5, *p* = 0.002) across conditions, these differences did not affect the main results.

**Experiment 3 Sample Demographics**

Data for Experiment 3 were collected from 16 December 2014 to 21 February 2015. A total of 759 individuals (*M*_age_ = 33.2, SD = 11.0 years; 50.5% females; Goodness-of-Fit *χ*² = 0.04, df = 1, *p* = 0.842) participated and were paid US$0.25. The raw sample included 796 participants of which 37 were dropped because they failed the audio-visual equipment check (*N* = 25) or did not complete the survey for undetermined reasons (*N* = 12). There was no significant difference in gender (Pearson *χ*² = 2.527, df = 5, *p* = 0.773), race/ethnicity (Fisher Exact *p* = 0.321), political views (Pearson *χ*² = 37.745, df = 30, *p* = 0.156), or annual income (Pearson *χ*² = 22.364, df = 20, *p* = 0.322) across conditions. Although there was a significant difference in age (Kruskal-Wallis *H* = 16.523, df = 5, *p* = 0.005), this difference did not affect the main results.

**Perception Measures between Video and Audio-Only Treatments**

**Experiment 1**

Participants in the audio-only treatments rated sharks significantly more negatively (*M* = 5.468) than those in the video treatments (*M* = 4.606; Mann-Whitney *U* = 62797.5, *p* < 0.001). Similarly, positive ratings were significantly lower in the audio-only treatments (*M* = 3.515) compared to the video treatments (*M* = 4.876; *U* = 22939, *p* < 0.001). Finally, the additional adjective provided by participants to describe sharks had significantly more negative valence in the audio-only conditions (*M* = -0.297) compared to the video treatments (*M* = 0.146; *U* = 30380.5, *p* < 0.001).

**Experiment 2**

There was no significant difference in negative ratings of sharks by participants in the audio-only treatments (*M* = 5.090) compared to those in the video treatments (*M* = 4.917; Mann-Whitney *U* = 86467.5, *p* = 0.110). However, positive ratings were significantly lower in the audio-only treatments (*M* = 4.090) compared to the video treatments (*M* = 4.441; *U* = 69317.5, *p* < 0.001). Lastly, the additional adjective provided by participants to describe sharks had significantly more negative valence in the audio-only conditions (*M* = -0.129) compared to the video treatments (*M* = 0.032; *U* = 60966.5, *p* = 0.017).

**Experiment 3**

Participants in the audio-only treatments rated sharks significantly more negatively (*M* = 5.249) than those in the video treatments (*M* = 4.445; Mann-Whitney *U* = 93262, *p* < 0.001). Similarly, positive ratings were significantly lower in the audio-only treatments (*M* = 3.920) compared to the video treatments (*M* = 5.065; *U* = 40986, *p* < 0.001). Finally, the additional adjective provided by participants to describe sharks had significantly more negative valence in the audio-only conditions (*M* = -0.219) compared to the video treatments (*M* = 0.263; *U* = 44861, *p* < 0.001).
